# Factors Influencing Non-Fungible Tokens (NFT) Game Engagement during the COVID-19 pandemic: The Theory of Planned Behavior (TPB) and Hedonic Motivation System Adoption Model (HMSAM) Approach

**DOI:** 10.1016/j.heliyon.2023.e19847

**Published:** 2023-09-04

**Authors:** William Davin D. Perez, Yogi Tri Prasetyo, Maela Madel L. Cahigas, Satria Fadil Persada, Michael Nayat Young, Reny Nadlifatin

**Affiliations:** a School of Industrial Engineering and Engineering Management, Mapúa University, 658 Muralla St., Intramuros, Manila, 1002, Philippines; b School of Graduate Studies, Mapua University, 658 Muralla St, Intramuros, Manila, 1002, Philippines; c International Bachelor Program in Engineering, Yuan Ze University, 135 Yuan-Tung Road, Chung-Li, 32003, Taiwan; d Department of Industrial Engineering and Management, Yuan Ze University, 135 Yuan-Tung Road, Chung-Li, 32003, Taiwan; e Entrepreneurship Department, BINUS Business School Undergraduate Program, Bina Nusantara University, Jakarta, 11480, Indonesia; f Department of Information Systems, Institut Teknologi Sepuluh Nopember, Kampus ITS Sukolilo, Surabaya, 60111, Indonesia

**Keywords:** Non-fungible token, Blockchain, NFT games, Theory of planned behavior, Hedonic motivation system adoption model, Structural equation modeling

## Abstract

The prominent form of Non-Fungible Token (NFT) is found in the gaming industry. NFT games received immense attention during the COVID-19 pandemic because of their play-to-earn model. NFT gamers can enjoy and increase their finances in their spare time. Hence, the researchers utilized Structural Equation Modeling (SEM) to investigate the intention and immersive behaviors of 1082 respondents. The modified framework from the Theory of Planned Behavior (TPB) and Hedonic Motivation System Adoption Model (HMSAM) underwent SEM tests. These theories and methods were used to analyze relationships among hypotheses and assess factors influencing NFT game engagement. The results showed that hedonic motivation produced positive and significant influences on perceived usefulness, curiosity, joy, attitude, subjective norms, and perceived behavioral control. Subjective norms significantly influenced perceived ease of use. In due course, perceived ease of use yielded positive and significant effects on perceived usefulness, joy, attitude, and perceived behavioral control. Moreover, perceived usefulness, curiosity, joy, attitude, and perceived behavioral control had significant positive effects on behavioral intention. In addition, perceived usefulness, curiosity, joy, and attitude significantly and positively affected immersion. Meanwhile, only four hypotheses were not supported by the study. These findings were translated into theoretical and managerial implications to contribute to the academe given the strong the change of behavior of users towards NFT games during the pandemic; gaming industry since they will be able to develop, improve and create a new ecosystem in the gaming space, and NFT stakeholders since they will benefit from the development that will influence this study.

## 1 Introduction and review of related literature

Non-Fungible tokens (NFTs) are unique digital assets, such as media content, artworks, photos, and videos and that have entered in the collectibles market [1]. Its popularity skyrocketed during the COVID-19 pandemic, resulting in a surge of market investors. The market growth rate increased by 115% at the end of 2020 [2]. These instances exhibited that consumers, middlemen, and businessmen benefited from the existence of NFTs. Thereby, the NFT huge market and influence as it can be accounted as an investment. Since the COVID-19 pandemic paved the way for digital maximization, NFTs attracted online trading between consumers and sellers in the absence of physical collectibles and the marketplace. It was supported that NFT was commonly merged with the gaming industry [3].

NFT has utilized the gaming sector to increase its market share. Moreover, the union of NFTs and the gaming industry attracted both investors and gamers. According to Bakhanova et al. [4], the process of gamifying a particular application increases user engagement due to the simplification of the context, motive, and overall objective of the system. Based on their findings, this is the most probable reason behind the soaring NFT games popularity.

NFT games are popular among Filipinos because of the play-to-earn model, where users have the privilege to enjoy the games while earning money. Axie Infinity, Mir4, and Pegaxy are some of the most played NFT games in the Philippines; thus, these NFT games are assessed in the current study. The COVID-19 pandemic made Filipinos look for other sources of income because of the increasing unemployment rate and living expenses [5]. Filipinos had the highest number of NFT asset ownerships (32%) among the identified twenty countries [5]. In addition, Axie Infinity gamers are mostly comprised of Filipinos compared to other countries [5]. Given the amount that needed to invest on the games played, there are lot of Filipinos value NFT games as an alternative investment than other markets such as forex, stock exchange, crypto and the likes [6]. Thus, the current study would like to assess the current behavior of Filipinos towards the popularity of NFT games as a way of earning money.

Despite the trending issue regarding NFT especially during the COVID-19 pandemic, factors affecting engagement in NFT games are still unclear. Bao & Roubaud [7] summarized relevant NFT studies and noted the corresponding methodologies, NFT types, and research fields. While those past studies frequently utilized empirical testing analogous to this present study, researchers commonly assessed general NFT's asset pricing. None of those collated past studies focused on the behaviors of Axie Infinity, Mir4, and Pegaxy gamers, which were assessed in the current research. One study examined NFT price behavior and users' trading volume during COVID-19 [8]. They assessed the top three NFTs in the global market, which represented the financial conditions of most NFT game users. However, the past study focused on NFTs' numerical standing and overlooked the importance of NFT users' behaviors. Although Francisco et al. [3] discussed Filipinos' perceptions of NFT games, they only utilized descriptive statistics. Francisco et al. [3] identified the possible risks and benefits of entering the market; volatility had the highest calculated risk, followed by financial stability. Trust issues in the platform's security, design, and usability were the primary concerns of the past study's respondents towards NFT games. Thus, their results were limited to the characteristics of data and they did not explore the underlying causes of NFT engagement issues.

In the current study, researchers generated an in-depth multivariate analysis of factors influencing NFT gamers. Yousaf & Yarovaya [9] disclosed the efficiency of herding behavior among NFT users; whereby, users tend to follow the decision of the crowd instead of following their personal investment analysis. This behavior was supported because of positive results as most NFT users maximized their financial returns by investing in recommended NFTs. However, the study only assessed one behavioral factor influencing NFT users. Therefore, several NFT engagement factors were taken into account in the present study. Meanwhile, Bharadwaj & Deka [10] explained the behavior of investors toward cryptocurrency and NFT games investment. The researchers combined the technology acceptance model and diffusion of innovation theory and utilized the SEM approach. They found that complexity, compatibility, and observability affected perceived ease of use and perceived usefulness, which influenced the overall behavioral intention of investors. However, investors have different behaviors compared to NFT game users. Therefore, the past study failed to cover NFT game users' behaviors. Furthermore, Zarifis & Castro [11] evaluated people's trust in purchasing NFT assets using structural equation modeling. They found that trust in cryptocurrency wallet, NFT marketplace, and dispute resolution are the significant variables. However, the past study did not employ any theories that could strengthen the findings. In the present study, two theories (theory of planned behavior and hedonic motivation system adoption model) are combined to fully assess NFT gamers' engagement.

Theory of planned behavior (TPB) is a goal-oriented approach as it analyzes the behavioral intention of a person driven by attitude, subjective norms, and perceived behavioral control [12]. The aforementioned variables pertain to the intrinsic motivation of a person [13]. Since the study intends to assess NFT gamers' engagement, their intrinsic motivations play a crucial role as they can be considered relevant factors. In this study, attitude reflects the insights of NFT gamers, subjective norms describe the influence of social pressure on NFT gamers, and perceived behavioral control refers to the capability and willingness of NFT gamers to perform the observed behavior. Arı et al. [14] explained gamers' behaviors using TPB. Although they failed to comprehensively adopt TPB since they replaced the standard variables with other motivational factors, their study supported that TPB could be utilized to identify factors influencing NFT gamers' engagement. Integration of theories happens because TPB is a generic model. TPB lacks specific motivational factors about NFT users’ cognitive engagement. Therefore, the researchers introduced specific variables by maximizing the concepts behind Hedonic Motivation System Adoption Model (HMSAM).

Hedonic Motivation System Adoption Model (HMSAM) is an acceptance model derived from the Technology Acceptance Model (TAM) and Hedonic Motivation System (HMS) [15]. It is inclined to underlying intrinsic motivations than extrinsic motivations since the model describes the facets of usability, pleasure, and enjoyment [16]. In the current research, 6 constructs from HMSAM are adopted in the integrated model. The following constructs are: (1) Perceived ease of use, (2) Perceived usefulness, (3) Curiosity, (4) Joy, (5) Behavioral intention, and (6) Immersion. The “control” construct from the original HMSAM was disregarded because perceived behavioral control from TPB better defined NFT gamers’ easiness or difficulty in performing the behavior [12,16]. Moreover, the behavioral intention from both HMSAM and TPB has similar implications and can be treated as one construct. Several studies noted that HMSAM is commonly used in gamification system and digital adoption [15,17,18] This instance supports the importance of HMSAM in the proposed model. Additionally, a sole “hedonic motivation” construct was introduced as one of the exogenous variables. Hedonic motivation describes the emerging positive emotions of a person [19]. Although it has similarities to the “joy” construct, the “hedonic motivation” construct is an exogenous variable that influences endogenous variables such as joy.

The current study determines the factors affecting Filipinos' intention and immersion to play NFT games during the COVID-19 pandemic. The researchers utilized SEM techniques and incorporated the concepts of the Hedonic Motivation System Adoption Model (HMSAM) and the Theory of Planned Behavior (TPB). These models are the key measures to find the underlying factors affecting NFT game users' engagement. The findings will be beneficial to NFT investors and companies since they can gauge user behaviors efficiently through the presented scientific and technical methods. Apart from NFT's direct stakeholders, the gaming and cryptocurrency industries can also utilize the study's results to improve and tailor their digital platforms based on the factors that will significantly affect immersion of users. In terms of the economy, the inclination of society to digitalization would likely increase the demand of NFTs. Thus, the research will also be able to pioneer the trend of disruption of digitalization to the economy. Lastly, the end beneficiary of this study would be the end users or players and the NFT artists. Through this study, they could expect that developments on the NFT games space would improve tremendously, as more research will start tackling NFTs.

## 2 Theoretical framework

**Fig. 1:**
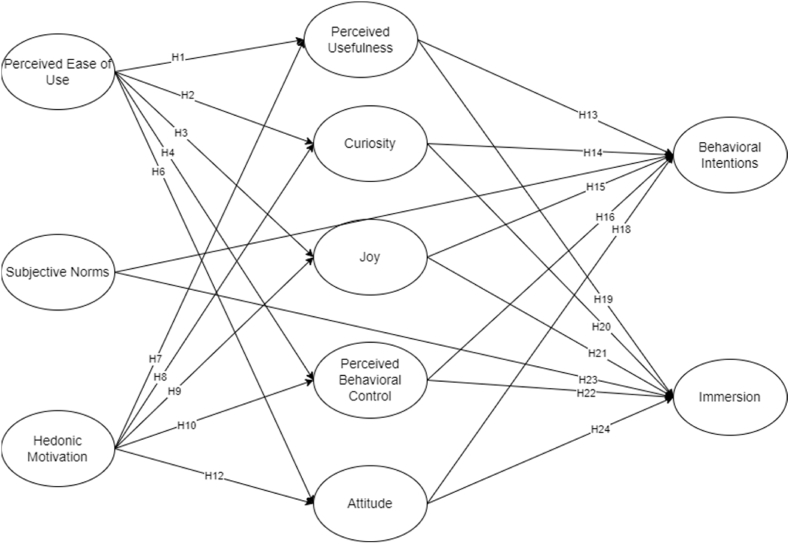
Theoretical framework.

The theoretical framework shown in Fig. 1 is grounded by the combination of both Theory of Planned Behavior (TBP) and Hedonic Motivation System Adoption Model (HMSAM). The following constructs are evaluated in the study: (1) Perceived Ease of Use (PEOU), (2) Subjective Norms (SN), (3) Hedonic Motivation (HM), (4) Perceived Usefulness (PU), (5) Curiosity (CUR), (6) Joy (JOY), (7) Attitude (ATT), (8) Perceived Behavioral Control (PBC), (9) Behavioral Intentions (BI), and (10) Immersion (IM). TPB primarily consists of subjective norms, attitude, perceived behavioral control, and behavioral intentions. Meanwhile, HMSAM comprises perceived ease of use, perceived usefulness, curiosity, joy, behavioral intentions, and immersion. As previously discussed, the hedonic motivation construct is introduced into the modified model.

Perceived ease of use acts as the core point of digital users to check if the system can be maneuvered effortlessly [15,17]. It determines the user's level of acceptance of the system's usefulness [20]. Digital applications are continuously developed to make things efficient as most people steer away from manual. Since digital users yearn for efficiency, applications that aren't difficult to use are preferred and considered useful [18]. In comparison to the current study, NFT gamers will most likely have a long-term commitment to games with a comprehensible manual. They would find it more useful to engage in games with simple instructions compared to complex ones. Another study disclosed that perceived ease of use produced a significant positive influence on perceived usefulness [15]. Therefore, the following hypothesis was generated.

Hypothesis 1 (H1) Perceived ease of use has a significant positive effect on perceived usefulness.

If games and digital tools are easy to use, there is a possibility that users will explore the game systems [21]. This instance coincides with the increase in user curiosity as discussed by Lowry et al. [16]. Past studies mentioned that the simplicity of games opens an opportunity to attract new users and maintain tenured users as engagement gradually increases [16,21]. However, the system's complexity is one of the initial barriers which hinders the user's curiosity [22]. This barrier creates a negative connection between NFT games and users because it reduces first-hand experience, enthusiasm, and curiosity. Thus, the researchers hypothesized that.

Hypothesis 2 (H2) Perceived ease of use has a significant positive effect on curiosity.

Quin Seng et al. [18] hypothesized that digital users enjoy the usage of applications if they can easily navigate the system. Hence, NFT gamers will enjoy the game if they deemed it easy to use. Although game interface has different elements, users value psychological reward (e.g., enjoyment) as long as ease of use is evident [15]. Furthermore, Lowry et al. [16] tested different sets of theoretical frameworks concerning the influence of perceived ease of use on joy. They found that all types of frameworks produce significant p-value. Meanwhile, Sun & Zhang [22] shared that perceived ease of use can be connected to joy in two directions. Their argument stated that joy influenced perceived ease of use only if utilitarian motivation is considered. However, the current study focuses on hedonic motivation since the researchers aim to analyze NFT gamers' emotions instead of the games' functionality. In addition, HMSAM concentrates on intrinsic motivations behind NFT gaming acceptance and engagement. Thus, the researchers formulated the constructs’ relationship based on the following hypothesis.

Hypothesis 3 (H3) Perceived ease of use has a significant positive effect on joy.

According to Kim et al. [23], the user's perceived ease of use of the technology system affects the user's attitude positively. Likewise, NFT games that are easy to use will attract more NFT users, resulting in a positive attitude. Other studies also suggested that perceived ease of use produced significant positive effects on attitude [24,25]. From a similar point of view, users who find convenience in using digital applications have an optimistic attitude, which returns favorable results [25]. On the contrary, complex applications instill a negative position in one's attitude. Thus, the researchers hypothesized that.

Hypothesis 4 (H4) Perceived ease of use has a significant positive effect on attitude.

Ease of system use will make people feel that they have full control of the digital platform as time passes by Ref. [26]. On the contrary, the difficulty increases if the system control is intricate. Similarly, Users feel at ease using newly introduced digital platforms if they are easy to use [27]. The past study supported that people value the convenience and flexibility of digital systems. As long as NFT games are easy to utilize, NFT gamers will find them easy to explore. These aforementioned concepts were also proven by the studies of Compeau & Higgins [28] and Agarwal & Karahanna [29]. Considering the pertinent studies, it was hypothesized that.

Hypothesis 5 (H5) Perceived ease of use has a significant positive effect on perceived behavioral control.

Subjective norms are the viewed pressures and obtained information from people within their environment [12]. In the current context, the researchers hypothesized that NFT gamers' peers and family members may affect engagement in NFT games. If users can acquire quality information, they can easily gauge the system's efficacy. The connection between the two constructs was tested by other researchers and deemed to have a significant positive relationship [25,30].

Hypothesis 6 (H6) Subjective norms have a significant positive effect on perceived ease of use.

Hedonic motivation refers to a person's intrinsic emotions and is usually associated with pleasure [19]. While hedonic motivation is used in different circumstances, a researcher revealed that it is more relevant in the technology sector [31]. NFT is a prominent token in blockchain technology. Its users are seemingly increasing during the COVID-19 pandemic. Hence, hedonic motivation further helps the investigation of factors influencing NFT user's engagement. Technology users show hedonism traits to utilizing useful digital applications [32]. The past study mentioned that interactive and immersive applications describe the usefulness of technology. Meanwhile, hedonic motivation includes curiosity and eventually affects hedonic motivation [33,34]. People are always curious-minded because they are eager to seek new experiences [19]. Thus, their motivations are driven by interesting and useful information [31,34]. Furthermore, hedonic motivation reflects positive experiences and emotions because people want to relieve stress [19]. As hedonic motivation increases, it is also posited that the feeling of joy is most likely to increase. Thus, the researchers established these hypotheses.

Hypothesis 7 (H7) Hedonic motivation has a significant positive effect on perceived usefulness.

Hypothesis 8 (H8) Hedonic motivation has a significant positive effect on curiosity.

Hypothesis 9 (H9) Hedonic motivation has a significant positive effect on joy.

Hedonic motivation recognizes the user's behavioral control over digital applications [34]. Apart from control, hedonic motivation opens a path to grasp the user's attention [34]. In connection with the study, NFT gamers' hedonism gives an option to users whether they wish to engage in NFT games or dismiss the thought. NFT gamers have complete control over their expected behavioral engagement. But then, Dwivedi et al. [32] disclosed that digital gaming continuously increases users' hedonic motivation, which positively affects gamers' attitudes. Additionally, NFT users communicate with other gamers through different online platforms. These platforms act as a virtual community sharing the same passions and interests. This online community engagement reflects the principles of subjective norms. To & Sung [34] noted that these virtual communities boost social practices. Given these pertinent studies, the following hypotheses were posited.

Hypothesis 10 (H10) Hedonic motivation has a significant positive effect on attitude.

Hypothesis 11 (H11) Hedonic motivation has a significant positive effect on subjective norms.

Hypothesis 12 (H12) Hedonic motivation has a significant positive effect on perceived behavioral control.

Perceived usefulness refers to the quality of an application for the users [15]. Ideally, NFT games must have greater advantages over negative repercussions before users label them as useful applications. If NFT games have several disadvantages, they would not be deemed useful for the users because users are only interested in using applications that would produce benefits. Likewise, perceived usefulness has a significant effect on behavioral intentions [35]. Although perceived usefulness has a limited impact on behavioral intention, it is still deemed a significant variable despite the constant modification of the original HMSAM [16]. The hypothesis was further supported by multiple research methods presented in past studies [36,37]. Hence, this hypothesis was made.

Hypothesis 13 (H13) Perceived usefulness has a significant positive effect on behavioral intentions.

Curiosity is both sensory and cognitive, resulting in human engagement [15,16]. When a user has developed a curiosity about the technology system, the user yields an intention to use it [38]. The past study also supported that curiosity has a mediating effect on behavioral intention. These past studies imply that once users of NFT games express curiosity, they are likely to use those applications. Since humans are bound to satisfy their curiosity, they intend to perform the expected behavior than leave it ignored [16]. Given these significant studies, this hypothesis was postulated.

Hypothesis 14 (H14) Curiosity has a significant positive effect on behavioral intentions.

Joy is defined as fun and enjoyable [15]. NFT gamers express different emotions when playing games. They feel happy when they win a specific battle, acquire unique digital items, or complete a specific level. On the other hand, some gamers display misery if they fail to accomplish game challenges. Between these two dissimilar emotions, joy or happiness resonates with users of technology systems [15,16]. Furthermore, users who feel joy easily accept the presented games [39,40]. Other studies also verified that joy created a significant effect on behavioral intentions [41,42]. In a nuthsell, the researchers suggested this hypothesis.

Hypothesis 15 (H15) Joy has a significant positive effect on behavioral intentions.

Perceived behavioral control, subjective norm, and attitude are the primary constructs influencing behavioral intention [12]. Perceived behavioral control refers to the person's resources and ability to perform the intended behavior [12]. In this case, resources include NFT games knowledge, internet connection, and device (mobile phone or computer). NFT gamers who have these resources find it easy to play online games compared to those who lack one of the resources [43]. Meanwhile, the subjective norm is the social pressure felt by the person [12]. NFT games have online communities where gamers interact with each other. NFT gamers' bond gets stronger as they share common interests in playing NFT games [43]. This in turn implies that virtual interaction may positively influence one's intention in playing NFT games. Since online communities tend to invite new users, people are more likely to follow their peers [43]. Lastly, attitude is a two-way path because humans can either reflect positive or negative emotions [44]. Humans' positive attitudes are displayed after demonstrating the reflective needs and advantages of technology systems [25]. Hence, NFT games that yield great returns will make gamers act positively in playing the games. But generational differences are considered when adopting a technology system [32]. It can be noted that young generations are mores inclined to play NFT games compared to the older generations. These related studies helped the assumption of the following hypotheses.

Hypothesis 16 (H16) Attitude has a significant positive effect on behavioral intentions.

Hypothesis 17 (H17) Subjective norm has a significant positive effect on behavioral intentions.

Hypothesis 18 (H18) Perceived behavioral control has a significant positive effect on behavioral intentions.

NFT games allow users to play the application individually or with a team. Since individual and multiplayer game interfaces vary, NFT gamers value digital presentation. This presentation comprises game characters, avatars, and emotional images [45]. These digital images influence the usefulness of NFT games because they trigger an association with NFT game engagement [45]. In this study, NFT game engagement implies immersion [15]. Online applications are defined as immersive if they offer comprehensive functions [32]. This shows that multifaceted-based NFT games attract potential gamers and retain current users. Another similar study noted the significant relationship between perceived usefulness and immersion [15]. Thereby, the researchers adopted the following hypothesis.

Hypothesis 19 (H19) Perceived usefulness has a significant positive on immersion.

Curiosity drives immersion in playing online games [45]. Gamers focus on NFT games if they want to unveil game secrecies and complete game challenges. The play-to-earn model entices users to explore NFT games, too. They are intrigued to further their skills and maximize their efforts to please their curiosity [46]. As a result, curious people take full advantage of resources (e.g., financial, physical, and knowledge) to meet their expectations and demonstrate their goals [16]. It was also confirmed that curious person disregards negative factors that are misaligned with their values [16,46]. This circumstance leads to immersion as negative factors are incapable to stop gamers from being immersed in playing NFT games. Moreover, Lowry et al. [16], hypothesized the relevant relationship between curiosity and immersion using HMSAM. With help of these past studies, the researchers suggested that.

Hypothesis 20 (H20) Curiosity has a significant positive on immersion.

Since NFT games offer both excitement and income, gamers are more inclined to play them constantly. However, technology applications must be adored by gamers before they fully commit themselves [43]. While NFT games allow users to earn money, pleasurable factors should not be overlooked. It must aim to fulfill the desire of users [45]. If not, NFT gamers will not excel in the games. The lack of enjoyment will cause failure to game challenges. If this happens, the feeling of joy is less likely to influence immersion [47]. Furthermore, it should be noted that gamers enjoy multiplayer competition in online games [47]. Winning in multiplayer challenges fulfills the player's hunger for joy and it eventually affects immersion. Considering these relevant studies, it was hypothesized that.

Hypothesis 21 (H21) Joy has a significant positive on immersion.

Most NFT games are developed intricately to increase user engagement. This factor keeps NFT gamers engaged because they are eager to overcome online missions through their abilities. However, users must have grit and adequate technical knowledge [44,47]. These abilities refer to one's perceived behavioral control. Online users who can overcome these controls in navigating NFT games are likely to feel immersed [16]. In addition, social influence is directly associated with subjective norms. Interacting with fellow gamers increases one's immersion [43]. This is referred to as an online community in the digital world. Members of the community easily connect because they share similar gaming interests [45]. The gamer can confide in another person quickly. A person may need help to solve NFT missions, seek advice to improve performance, and talk about interests. However, all NFT gamers have their corresponding gaming performance and learning curve. A study noted that a gamer prefers meeting an equally good or well-performing gamer to the underperforming one [47]. This past study discussed the importance of knowledge sharing among gamers at a similar or higher level as it increases immersion. If NFT gamers nurture their knowledge, it will also allow them to excel in the game and earn more. Finally, a person's positive attitude reflects an immersion in the digital application because they inflict a positive state of mind and dedicate attention [16,45]. While there are negative connotations such as addiction and physical disconnection, one's attitude is entirely dependent on the person [46]. This scenario happens in NFT gaming frequently due to the play-to-earn structure. As a matter of course, attitude is one of the strongest exogenous TPB variables [12]. Users' attitudes primarily consist of individual principles and experiences [32]. Therefore, the following hypotheses were generated.

Hypothesis 22 (H22) Attitude has a significant positive on immersion.

Hypothesis 23 (H23) Subjective norm has a significant positive on immersion.

Hypothesis 24 (H24) Perceived behavioral control has a significant positive on immersion.

## 3 Methodology

The researchers analyzed factors influencing NFT game engagement using theoretical and statistical analysis called structural equation modeling. In this section, the methodology comprised three sections. First, the researchers discussed the respondents’ demographic characteristics. Second, the Google Form questionnaire was presented. Third, the principles behind structural equation modeling were elaborated. This study was approved by Mapua University Research Ethics Committees (FM-RC-23-07) and an informed consent was obtained from all participants prior to the data collection.

### 3.1 Respondents

The researcher utilized a purposive sampling technique to collate the participants’ responses from January 13 to January 27, 2022. This is an efficient technique as it aims specific respondent characteristics [13]. All participants experienced playing NFT games, which verifies the reliability of the data collection. The survey process was done through social media platforms and was directly disseminated to specific groups and pages that primarily discuss the NFT games to ensure that participants are fit to answer the survey. Moreover, the researcher contacted and requested several group administrators and leaders to encourage their members to answer the survey. Hence, the researcher was able to get 1082 participants. Table 1 shows the diverse demographic characteristics of 1082 respondents. The details were beneficial to assess the behavior of different groups engaged in NFT games.

**Table 1:** Demographic profile.

Characteristics	Category	N	Percentage
Age Group	Generation Z (1997–2003)	878	81.15%
	Millennial (1981–1996)	198	18.30%
	Generation X (1965–1980)	6	0.55%
Gender	Male	823	76.10%
	Female	259	23.90%
Occupation	Student	705	65.16%
	Employed	141	13.02%
	Self-Employed	74	6.84%
	Unemployed	162	14.98%
Monthly Income	≤ ₱20,000	970	89.60%
	> ₱20,000, < ₱40,000	73	6.70%
	> ₱ 40,000, < ₱ 60,0000	16	1.50%
	> ₱ 60,000 to < ₱ 80,000	12	1.10%
	> ₱ 80,000, < ₱ 100,000	5	0.50%
	≥ ₱100,000	6	0.60%
Purpose of Playing NFT Games	Passive Source of Income	621	57.40%
	Primary Source of Income	461	42.60%
Period of Playing NFT Games	During the COVID-19 pandemic	1006	92.98%
	Before the COVID-19 pandemic	76	7.02%

Out of the 1082 respondents, 81.15% of respondents were part of the Generation Z age group which were born on year 1997 till 2003, 18.30% were Millennials, born on 1981 up to 1996, and only 0.55% belonged to the Generation X, which are born from 1965 to 1980. The questionnaire was dominated by male (76.10%) compared to female (23.90%) respondents. This circumstance was plausible because gaming was a male-dominated industry. Meanwhile, more than half of the respondents were students (65.16%), 14.98% were unemployed, 13.02% were full-time employees, and 6.84% were self-employed. Their careers coincided with the age groups since most of the respondents were in the younger generation. Moreover, students and unemployed individuals don't have immense commitment, which urges them to explore NFT games. Also, a lot of respondents (89.60%) had the least monthly income (at most ₱20,000, approximately $360) because the corresponding age groups and occupations were known for limited financial resources. A combined 10.40% of total respondents earned more than ₱20,000. Interestingly, 57.40% of respondents used NFT games as a passive source of income and the remaining respondents (42.60%) perceived them as a primary source of income. The increase in NFT users during the COVID-19 pandemic was supported in the study because 92.98% of the study's NFT gamers started playing during the pandemic compared to the pre-pandemic period (7.02%).

### 3.2 Questionnaire

The data collection medium was online based due to the COVID-19 constraints of conducting face-to-face interaction. The researchers utilized different social media platforms (Facebook, Instagram, Twitter) commonly used by the public. The Google Form was disseminated by posting on online groups publicly. All respondents were deemed to have experienced playing NFT games.

The questionnaire for the integrated HMSAM and TPB was developed using relevant studies as shown in Table 2. A 5-point Likert scale was used with a measure of “Strongly Agree” at a rate of 5 to “Strongly Disagree” at a rate of 1. HMSAM's purpose is to assess the intrinsic motivations that affect the acceptance and engagement of users to NFT games [16]. Meanwhile, TPB tackles the importance of attitude, subjective norms, and perceived behavioral control to explain the behavioral intention of the person towards NFT games [12].

**Table 2:** The construct and measurement items.

Construct	Code	Indicator	Reference
*Perceived Ease of Use*	PEOU1	My interaction with the game was clear and understandable.	Lowry et al. [16],
	PEOU2	Interacting with the game did not require a lot of mental effort.	
	PEOU3	I found the game to be trouble-free.	
	PEOU4	Learning to operate the game was easy for me.	
	PEOU5	It was simple to do what I wanted with the game.	
	PEOU6	It was easy for me to excel at playing the game.	
	PEOU7	I found the game easy to use.	
*Hedonic Motivation*	HM1	Playing NFT games is one of my favorite activities.	Changa et al. [48],
	HM2	I am a person who is looking for more fun and enjoyment in NFT games.	Changa et al. [48],
	HM3	I feel pleased with the adoption of NFT games.	Salimon et al. [49],
	HM4	I can forget my problems when playing NFT games.	Lee [50]
*Perceived Usefulness*	PU1	The game decreased my stress.	Lowry et al. [16],
	PU2	The game helped me better pass the time.	Lowry et al. [16],
	PU3	The game provided a useful escape.	Lowry et al. [16],
	PU4	The game helped me think more clearly.	Lowry et al. [16],
	PU5	The game helped me feel rejuvenated.	Lowry et al. [16],
	PU6	The game enhanced my productivity.	Oluwajana et al. [15]
	PU7	I find NFT games as a useful tool for earning income.	Oluwajana et al. [15]
	PU8	NFT games enhance my effectiveness in earning income.	Agarwal & Karahanna [29]
*Curiosity*	CUR1	Playing NFT games excites my curiosity.	Lowry et al. [16],
	CUR2	NFT games make me curious.	
	CUR3	NFT games aroused my imagination.	
*Joy*	JOY1	I found playing the game to be enjoyable.	Lowry et al. [16],
	JOY2	I had fun playing the game.	
	JOY3	I never felt bored playing the game.	
	JOY4	The game did not annoy me.	
	JOY5	I felt satisfied playing the game.	
*Perceived Behavioral Control*	PBC1	I have the knowledge to play NFT games.	Lee & Tsai [51]
	PBC2	I have the resources to play NFT games.	Lee & Tsai [51]
	PBC3	I have the ability to play NFT games.	Lee & Tsai [51]
	PBC4	I can easily and readily access information about NFT game functions.	Ong et al. [52]
	PBC5	I believe that whether or not I will play NFT games is entirely up to me.	Ong et al. [52]
*Subjective Norm*	SN1	People who are important to me supported my decision to play NFT games.	Lee & Tsai [51]
	SN2	People who influence my behavior wanted me to play NFT games instead of alternative activities.	Lee & Tsai [51]
	SN3	Most people who are important to me would think that playing NFT games is a wise idea.	Lee & Tsai [51]
	SN4	If people around me play NFT games, I will also do the same thing.	Ong et al. [52]
*Attitude*	ATT1	I think it's easy to play NFT games.	Ong et al. [52]
	ATT2	I think playing NFT games is convenient.	Ong et al. [52]
	ATT3	I feel relaxed every time I play NFT games.	Ong et al. [52]
	ATT4	I think playing NFT games is a responsibility.	Ong et al. [52]
	ATT5	I think playing NFT games is … (Harmful: Beneficial)	Finke et al. [53]
	ATT6	I think playing NFT games is … (Risky: Safe)	Finke et al. [53]
	ATT7	I think playing NFT games is … (Foolish: Wise)	Finke et al. [53]
*Behavioral Intention*	BI1	I intend to play more NFT games in the future.	Oluwajana et al. [15]
	BI2	I intend to continue playing NFT games.	Oluwajana et al. [15]
	BI3	I expect to play NFT games in the future.	Oluwajana et al. [15]
	BI4	NFT games make earning income more interesting.	Oluwajana et al. [15]
	BI5	I like the idea of playing NFT games.	Oluwajana et al. [15]
	BI6	I intend to use NFT games as a source of income.	Ong et al. [52]
*Immersion*	IM1	I blocked notable distractions while playing the game.	Oluwajana et al. [15] Mulaji et al. [54] Khan et al. [55]
	IM2	I was absorbed in what I was doing.	
	IM3	I was immersed in the game.	
	IM4	I was not interrupted by unforeseen obstructions very easily.	
	IM5	My attention was not diverted very easily.	

### 3.3 Structural equation modeling

The researchers employed Structural Equation Modeling (SEM) through SmartPLS 3. SEM’S confirmatory factor analysis (CFA) was considered since measures are pre-established and theories exist beforehand [56]. This technique is more suitable for theoretical framework development [[57], [58], [59]]. Partial Least Squares-SEM approach is a commonly used technique to calculate the relationship using total variance and predictive modeling. Thus, PLS-SEM can perform exploratory and confirmatory research, given its strong capability solving small or large sample size and even with non-normalized data [[60], [61], [62], [63]]. The researchers adopted two existing theories (HMSAM and TPB) and introduced a separate hedonic motivation latent construct to complete the proposed model. All these ideologies were merged to gauge variables influencing NFT gamers' engagement.

This study evaluated a total of 10 latent variables or constructs: (1) Perceived ease of use, (2) Subjective norms, (3) Hedonic Motivation, (4) Perceived usefulness, (5) Curiosity, (6) Joy, (7) Attitude, (8) Perceived behavioral control, (9) Behavioral intentions, and (10) Immersion. Each construct comprised 3 to 8 measures or indicators, which described the constructs clearly. As a result, a total of 54 indicators were investigated. All constructs and indicators underwent SEM data consistency metrics to ensure reliability.

Structural equation modeling must meet several quality criteria to ensure that the model is deemed valid. According to Hair et al. [61], the model must pass several statistic measurements to assess model validity respectively which are: indicator and factor loadings are, construct validity and reliability, R-square, discriminant validity, collinearity statistic, and the model fit. Indicator and factor loading interprets the given indicators and construct are acceptable and reliable. Meanwhile, construct validity and reliability verify the internal consistency of each construct. R-square provides the explanatory power which shows prediction strength of each endogenous constructs. Discriminant validity is a calculation indicating the distinction of each construct. Collinearity statistics show the measure of correlation between two independent variables, this ensures that each construct does not show multicollinearity which affects accuracy of the predictor variables. Lastly, model fit is the assessment used for measuring residuals and deviations of covariances that may impact the suitability of each construct as a whole model. This assessment is commonly measured through badness or goodness of fit such as the Standardized Root Mean Residual (SRMR) and Normal Fit Index (NFI) [64]. Hence, the said assessments and criteria of the SEM will be used in the current study.

## 4 Results

### 4.1 NFT game player attributes

**Fig. 2:**
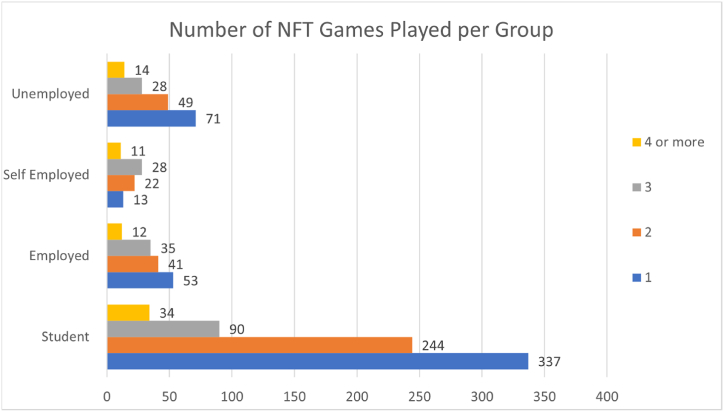
Number of NFT games played per group.

Fig. 2 shows the number of NFT games played by four distinct groups. 48% of the students only play one (1) NFT game while, 38% for employed, 18% for self-employed, and 44% for the unemployed group. Meanwhile, 35% of students are playing two (2) NFT games 29% for employed, and 30% for both self-employed and unemployed groups. 13% of the students are playing 3 NFT games while 25%, 38%, and 17% of the employed, self-employed, and unemployed, respectively are engaged with 3 NFT games. Lastly, both the employed and unemployed got 9% of their count plays 4 or more NFT games while only 11 of 74 self-employed and 34 of 705 students play 4 NFT games.

**Fig. 3:**
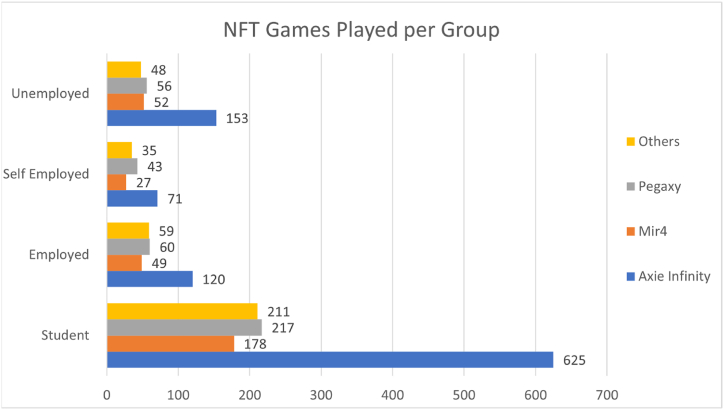
NFT games player per group.

Fig. 3 shows that Based on the data collected from the respondents, it seems that the occupation of individuals affects their engagement with NFT games. Among the surveyed population, students accounted for the largest group, with 705 respondents. Within this group, Axie Infinity was the most popular NFT game, played by 625 students. Mir4 and Pegaxy followed, with 178 and 217 student players respectively. Other NFT games were played by 211 students. Unemployed individuals comprised the second largest group, with 162 respondents. Among them, 153 played Axie Infinity, while Mir4 and Pegaxy had 52 and 56 players respectively. Other NFT games were played by 48 unemployed individuals. The self-employed group consisted of 74 respondents. Axie Infinity remained the most popular game in this group as well, with 71 players. Mir4 and Pegaxy had 27 and 43 players respectively, and other NFT games were played by 35 self-employed individuals. Lastly, the employed group included 141 respondents. Axie Infinity continued to be the most played NFT game in this category, with 120 players. Mir4 and Pegaxy followed, with 49 and 60 employed players respectively. Other NFT games were played by 59 individuals with employment.

### 4.2 Indicator and factor loadings

**Fig. 4:**
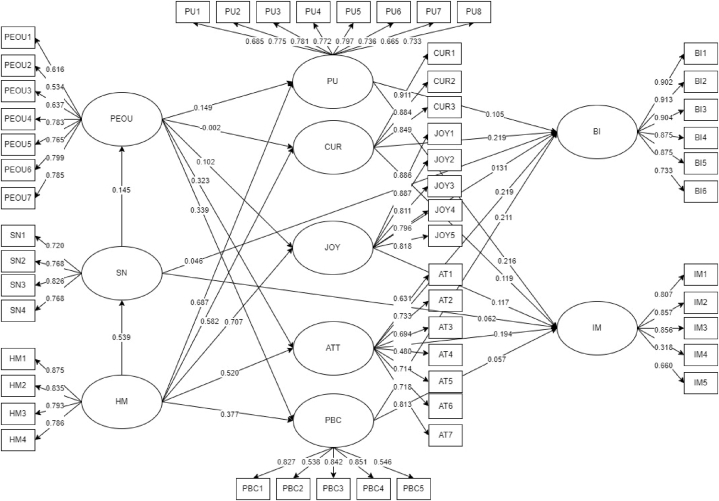
Initial SEM result.

Fig. 4 displays the initial values of factor loadings and direct coefficients. Factor loadings denote indicator values (e.g., PEOU1: 0.616, PEOU2: 0.534, PEOU3: 0.637) reflected from their corresponding constructs. Based on the initial result, factor loadings were at least 0.318. Additionally, direct effects between hypotheses (e.g., PEOU - > PU: 0149, PEOU - > CUR: 0.002, PEOU - > JOY: 0.102) comprised negative and positive values. The values ranged from −0.002 to 0.707. Positive values meant a similar direction while negative values meant the opposite direction as the proposed hypothesis.

**Fig. 5:**
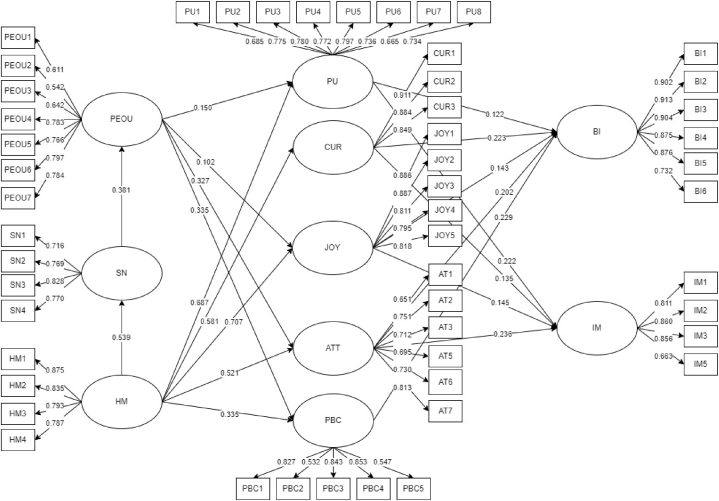
Modified SEM

Since some data consistency metrics in the initial result failed to meet the minimum cut-off, the framework must be modified by removing the failed paths and indicators [65]. The said modification will improve the overall accuracy and reliability of the model. First, factor loadings of indicators should be at least 0.50 [66]. AT4 and IM4 had a factor loading of 0.480 and 0.318, respectively. These indicators were eliminated in the modified model because they were deemed an unacceptable SEM measure. Next, there were no constraints for direct effects but their corresponding p-values must be less than 0.05 to indicate significant hypothesis relationships [66]. Unfortunately, four (4) hypotheses failed to meet the given parameters. Specifically, the relationships between PEOU - > CUR, SN - > BI, PBC - > IM, and SN - > IM had p-values of 0.955, 0.152, 0.080, and 0.072, respectively. Since their p-values were at least 0.05, they were eliminated in the modified model. Table 3 shows the hypotheses’ direct effects and p-values comparison between the initial and modified models. The modified model displayed better values and all hypotheses met the minimum 0.05 significant value. Moreover, they surpassed the minimum cut-off as all p-values were less than 0.01, which meant greater significance points. Finally, the modified and final framework is shown in Fig. 5.

**Table 3:** Relationship between hypothesis.

Hypothesis		Initial Model		Modified Model	
Number	Path	Effect (β)	P-value	Effect (β)	P-value
1	PEOU - > PU	0.149	0.001	0.150	0.001
2	PEOU - > CUR	−0.002	0.955	–	–
3	PEOU - > JOY	0.102	0.001	0.102	0.001
4	PEOU - > ATT	0.323	0.001	0.327	0.001
5	PEOU - > PBC	0.339	0.001	0.335	0.001
6	SN - > PEOU	0.381	0.001	0.381	0.001
7	HM - > PU	0.687	0.001	0.687	0.001
8	HM - > CUR	0.582	0.001	0.581	0.001
9	HM - > JOY	0.707	0.001	0.707	0.001
10	HM - > ATT	0.520	0.001	0.521	0.001
11	HM - > SN	0.539	0.001	0.539	0.001
12	HM - > PBC	0.377	0.001	0.379	0.001
13	PU - > BI	0.105	0.012	0.122	0.002
14	CUR - > BI	0.219	0.001	0.223	0.001
15	JOY - > BI	0.131	0.007	0.143	0.004
16	ATT - > BI	0.219	0.001	0.202	0.001
17	SN - > BI	0.046	0.152	–	–
18	PBC - > BI	0.211	0.001	0.229	0.002
19	PU - > IM	0.216	0.001	0.222	0.001
20	CUR - > IM	0.119	0.001	0.135	0.001
21	JOY - > IM	0.117	0.010	0.145	0.002
22	ATT - > IM	0.194	0.001	0.236	0.001
23	SN - > IM	0.062	0.072	–	–
24	PBC - > IM	0.057	0.080	–	–

### 4.3 Construct reliability and validity

After the removal of unacceptable constructs and indicators, data reliability was determined as shown in Table 4. The constructs' validity was measured through Cronbach's alpha, composite reliability, and Average Variance Extracted (AVE). The rule of thumb for both Cronbach's alpha and composite reliability is to have a value of at least 0.70 [67]. Based on findings, all the values of Cronbach's alpha and composite reliability were at least 0.70. Cronbach's alpha ranged from 0.773 to 0.934. Meanwhile, the constructs' composite reliability values met the minimum 0.70 cut-off. These values were between 0.860 and 0.949. Therefore, the constructs' Cronbach's alpha and composite reliability values depicted internal consistency. To check for the convergent validity of constructs, it must be observed through AVE. Hair et al. (2020) stated that the standard measure for AVE is at least 0.50 [67]. All constructs yielded more than 0.50 AVE values.

**Table 4:** Constructs’ reliability and validity.

Construct	Cronbach's Alpha	Composite Reliability	AVE
PEOU	0.833	0.875	0.504
SN	0.773	0.854	0.595
HM	0.84	0.893	0.677
PU	0.884	0.908	0.554
CUR	0.854	0.911	0.774
JOY	0.895	0.923	0.706
ATT	0.821	0.87	0.528
PBC	0.778	0.85	0.541
BI	0.934	0.949	0.756
IM	0.811	0.877	0.642

### 4.4 R-square

On the other hand, Table 5 shows the R-squared values of endogenous variables from the modified model. Among the ten constructs, nine are endogenous (PEOU, SN, PU, CUR, JOY ATT, PBC, BI, IM) and only one is considered exogenous (HM). Endogenous variables or constructs are influenced by other variables and exogenous variables are not influenced by other variables [68]. As seen in the abovementioned frameworks, HM is the sole construct that isn't affected by any of the nine constructs. Meanwhile, the R-squared value is the magnitude of variance presented by endogenous variables influenced by their corresponding exogenous variables. According to Hair [68], the R-squared value ranges from 0 to 1 and there is no standard threshold. The modified model's r-squared values ranged from 0.145 to 0.586. Values that are closer to 1 signify the best result [13]. For instance, PU accounted 58.6% data fit as it had the highest R-squared value/while PEOU had 14.5% data fit only. This meant that PU was best explained by its connected exogenous variables compared to PEOU.

**Table 5:** R-Squared values of endogenous constructs.

Endogenous Construct	Initial Model	Modified Model
PEOU	0.145	0.145
SN	0.291	0.291
PU	0.586	0.586
CUR	0.338	0.337
JOY	0.575	0.575
ATT	0.527	0.532
PBC	0.372	0.371
BI	0.56	0.553
IM	0.405	0.406

### 4.5 Discriminant validity

Discriminant validity can be analyzed through Fornell-Lacker criterion analysis and Heterotrait-Monotrait (HTMT) ratio. According to Hair et al. [67] and Henseler et al. [69], HTMT ratio is a better tool than Fornell-Lacker to guarantee the model's validity. Several studies mentioned that values should be less than 0.90 [[69], [70], [71]]. As seen in Table 6, all constructs yielded within the given threshold. Thereby, the correlation between constructs is reliable.

**Table 6:** Heterotrait-monotrait ratio.

Construct	ATT	BI	CUR	HM	IM	JOY	PBC	PEOU	PU	SN
ATT										
BI	0.679									
CUR	0.547	0.647								
HM	0.794	0.700	0.681							
IM	0.674	0.54	0.555	0.671						
JOY	0.766	0.689	0.700	0.867	0.657					
PBC	0.669	0.648	0.517	0.629	0.514	0.612				
PEOU	0.687	0.363	0.285	0.517	0.440	0.476	0.591			
PU	0.823	0.684	0.678	0.873	0.690	0.891	0.579	0.527		
SN	0.762	0.586	0.488	0.661	0.557	0.653	0.598	0.461	0.679	

### 4.6 Collinearity Statistic

Measuring collinearity can be calculated by obtaining the Variance Inflation Factor (VIF). A rule of thumb is that VIF values must be between 0.25 and 5 to ensure that collinearity is acceptable [72]. Looking at Table 7, all the values are within the acceptable range. Thereby, it is expected that all constructs collinearity is acceptable.

**Table 7:** Collinearity statistic - VIF.

Construct	AT	BI	CUR	HM	IM	JOY	PBC	PEOU	PU	SN
AT		2.329			2.141					
BI										
CUR		1.736			1.700					
HM	1.253		1.000			1.253	1.253		1.253	1.00
IM										
JOY		3.153			3.084					
PBC		1.581								
PEOU	1.253					1.253	1.253		1.253	
PU		3.338			3.338					
SN								1.000		

### 4.7 Model fit

The fitness of the model shown in Table 8 was assessed based on the given Standard Root Mean Square Residuals (SRMR) and Normed-Fit Index (NFI). As for the SRMR threshold established by Hu & Bentler [73], 0.00 to 0.08 value determines the discrepancies within the model. Meanwhile, the NFI threshold established by Oke et al. [74] must range from 0.60 to 1.00. This value represents the model's suitability depending on the deviation from the absolute fit model. The computed values for the modified model's SRMR and NFI were 0.07 and 0.80, which were within the standard threshold.

**Table 8:** SEM fit indices.

Fit Index	Initial Model	Modified Model	Threshold	Reference
SRMR	0.07	0.07	0.00 to 0.08	Hu & Bentler [73]
NFI	0.77	0.80	0.60 to 1.00	Oke et al. [74],

## 5 Discussion

### 5.1 Interpretation of SEM results

**Fig. 6:**
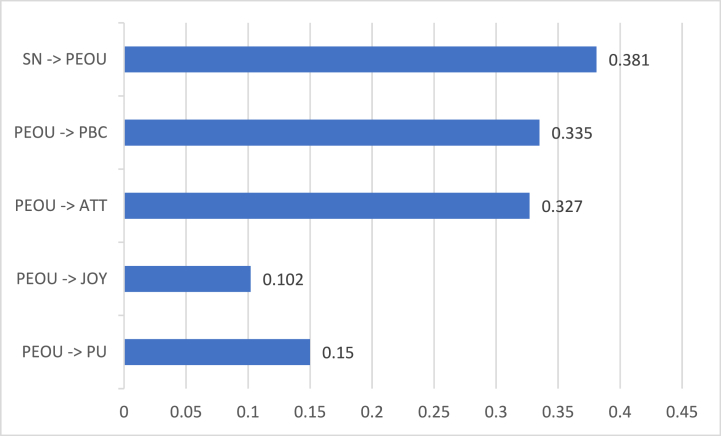
Hypotheses' direct coefficients involving perceived ease of use.

Fig. 6 shows the direct coefficients that involves PEOU obtained from the modified SEM column at Table 3. PEOU produced a positive and significant influence on PU (β = 0.15 and p = 0.001). NFT gamers who found it easy to play the game increased their productivity. Trouble-free games maximized the gamers' time in earning money and learning from the game. In the study of Oluwajana et al. [15], PEOU was the strongest predictor among all the constructs influencing PU. But in the current study, PEOU was the weakest predictor of PU because HM had a higher direct coefficient. This difference occurred because Oluwajana et al. [15] gauged students' online learning materials compared to NFT games that didn't entail any school grades. Nonetheless, this current study argued that earning money was a deciding factor during the COVID-19 pandemic as the unemployment rate increased. Second, PEOU had a positive significant influence on JOY (β = 0.102 and p = 0.001). NFT gamers enjoyed playing the games when simple instructions were provided. They didn't mind exploring NFT games as long as they were easy to play. Similarly, users must understand the online application's concepts before users enjoy using the application [22]. It was supported that PEOU produced a significant effect on JOY despite the several framework modifications [16]. Next, PEOU had a positive significant influence on ATT (β = 0.327 and p = 0.001) and PBC (β = 0.335 and p = 0.001). NFT gamers gave higher importance to ATT and PBC compared to PU and JOY. This posited that NFT games affect the principles, beliefs, and capabilities of users more than the usefulness and feeling of enjoyment. Gamers value their time, physical resources, and knowledge when engaging in NFT games. Park & Chung [45] also noted that the technical interface reflects the gamer's identity. Thus, users who could relate to the instructions and game interface were connected to the person's attitude and control.

However, PEOU did not produce any significant influence on CUR. Therefore, there was a lack of evidence between NFT games' trouble-free systems with the user's curiosity. This finding implied that PEOU was mostly associated with the user's actual perception than the assumption stage. PU, JOY, ATT, and PBC were considered the actual evaluation upon playing the game. Meanwhile, curiosity took place before playing the game. Curiosity is better used as an influencing factor to understand the succeeding action, such as curiosity to behavioral intention [46]. Although there was a study that disclosed a significant effect between the two constructs [16], it could be argued that the past study approached their SEM in an exploratory format while the current study used confirmatory.

SN significantly and positively affected PEOU (β = 0.381 and p = 0.001). The environment affected users' perceptions of the game's efficiency. The people surrounding the gamers helped them understand the game's operations and goals. Without the support of their peers, they would not find the game easy to use. Another study noted that the sociological aspects of gaming and cryptocurrency applications held immense power [32]. This includes the involvement of online communities in NFT gamers' lives. Likewise, online communities encouraged the use of NFTs since it is considered an investment [1]. Users who play and understand NFT games share their knowledge, resulting in a user-friendly interface.

**Fig. 7:**
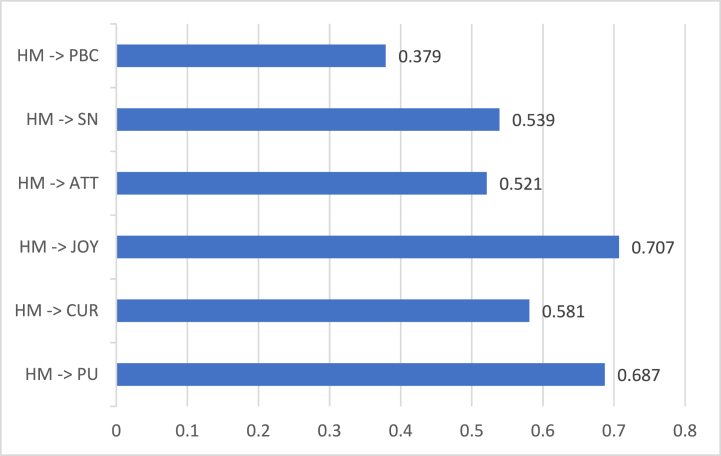
Hedonic Motivation's direct coefficients on corresponding constructs.

Furthermore, HM produced a positive and significant influence on PU (β = 0.687 and p = 0.001), CUR (β = 0.581 and p = 0.001), and JOY (β = 0.707 and p = 0.001) as shown in Fig. 7 obtained from the modified SEM column at Table 3. NFT gamers' psychological emotions and motivations were satisfied when NFT games were found useful, triggered their imagination, and made them enjoy. Their motivation to engage in the game relies on NFT games' benefits and their emotions. A past study disclosed that hedonic motivation increases if digital applications offer an interactive experience [32]. In the NFT games context, users' motivations improved due to the play-to-earn model. This posited that NFT games utilized excellent strategies in combining pleasure and financial aspects. In addition, Lowry et al. [16] suggested the inclusion of HM construct in HMSAM to analyze the original framework further. In the current study, HM was supplemented in the proposed framework and connected to HMSAM and TPB constructs. The results showed that HM had a greater effect on HMSAM constructs (PU, CUR, and JOY) compared to TPB constructs (ATT, SN, PBC). This implied that hedonic motivation held a greater effect on NFT games' usefulness and users' psychological emotions than the gamers’ beliefs and environment.

Next, Fig. 7 also shows that HM had a positive and significant effect on ATT (β = 0.521 and p = 0.001), SN (β = 0.539 and p = 0.001), and PBC (β = 0.379 and p = 0.001). Among all the primary TPB constructs, SN had the highest direct effect, followed by ATT, and PBC. These findings described that the hedonic motivation of gamers greatly influenced their acceptance of peer perceptions. Influences from peers yielded better effects compared to the gamer's actions and control. Yousaf & Yarovaya [9] disproved peer pressure's importance among NFT investors because of marketing instability. These investors prefer doing their own analysis to following others' perceptions. The current study argued that NFT investment has a different context compared to NFT games. NFT investment does not entail a play-to-earn model because it offers a pure financial interface, while NFT games aim to provide fun and earnings. In this study, gamers benefit from others by listening to the people surrounding them.

**Fig. 8:**
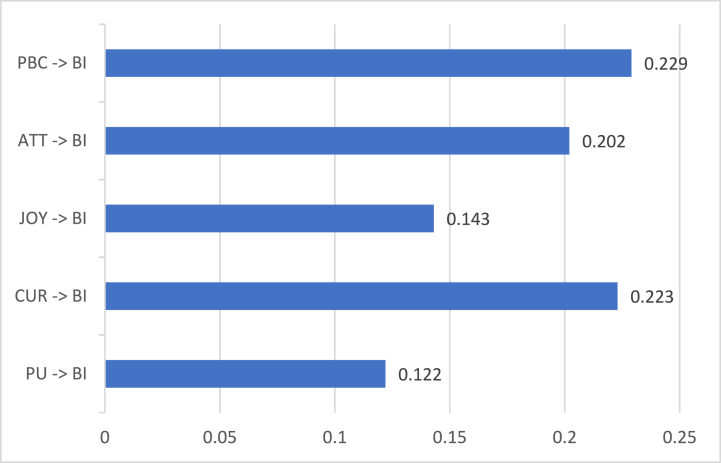
Hmsam and TPB constructs' direct coefficients on behavioral intentions.

Fig. 8 shows the direct coefficients involving BI obtained from the modified SEM column at Table 3. It is observed that PU (β = 0.122 and p = 0.002), CUR (β = 0.223 and p = 0.001), and JOY (β = 0.143 and p = 0.004) generated positive and significant effects on BI. Gamers intend to play NFT games in the long run if play-to-earn operations are consistent, they constantly ignite curiosity and imagination, and they felt joy and satisfaction while playing. On the other hand, NFT gamers would lose their intention to play if the games would produce disadvantages, such as losing money without recovering the principal amount. Also, they did not like games that would not exercise their minds. Lastly, NFT gamers did not want a mundane game. Similar to the findings of a past study, users wanted to ease boredom and feel excitement when playing games [43]. Another study concluded that the usefulness of games directly affects the user's intention to play the game [48]. Among the HMSAM constructs, CUR had the highest direct coefficient. This suggested that NFT gamers' curiosity produced a greater influence on behavioral intention than the game's usefulness and gamers' enjoyment. Thus, NFT gamers prioritized looking for games that stimulated their interests and offered uniqueness. These results were similar to the findings of Lowry et al. [16], Oluwajana et al. [15], and Park et al. [38].

The two TPB constructs (ATT and PBC) were found to have significantly affected NFT gamers’ intention to play. Specifically, ATT (β = 0.202 and p = 0.001) and PBC (β = 0.229 and p = 0.002) yielded positive and significant effects on BI. Since most respondents are from Generation Z, they are most likely inclined to try technological trends. Also, more than half of the participants do not have a stable income. Thus, apart from enjoying their youth, they also wanted to increase their financial resources. The current findings were similar to the study of Alzahrani et al. [43], where most respondents are students. They deemed that engaging in games was more favorable. Furthermore, NFT gamers believe in their ability to play NFT games. Since the generation is taken into consideration, Generation Z is more adept at online gaming. They can easily understand instructions and maneuver digital interfaces compared to the older generation. Likewise, PBC had a significant and positive influence on playing online games [43].

Unfortunately, SN did not create a significant impact on BI. This area had a contrasting result with the findings of Alzahrani et al. [43]. In this study, SN had only indirectly influenced BI through PEOU. However, peers' influence was not a strong factor to affect users' intention to play the games for a long period. The most convincing reason is the inclusion of spending money before venturing into NFT games. Engaging in play-to-earn-model needs capital as gamers had to invest in characters and skills. Although peers could help the users by giving advice and leveraging resources, the outcome is still dependent on the actual NFT gamer user. Another study disclosed that gamers tend to compare themselves to other gamers, may it be a worse or a better player [47]. This is associated with subjective norms and it produced a significant influence on behavioral intention. However, the subjective norm assessed in the current study was centered on the environment's influence to convince a person. Therefore, the competition facet was eradicated from evaluating respondents' subjective norms.

**Fig. 9:**
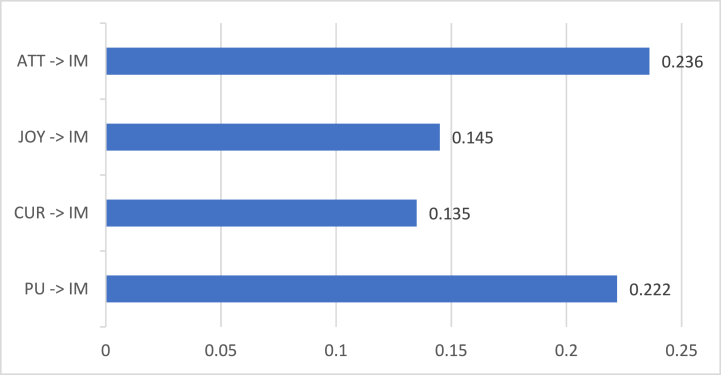
Hmsam and TPB constructs' direct coefficients on immersion.

Meanwhile, Fig. 9 shows the direct coefficients involving IM obtained from the modified SEM column at Table 3. Result shows that PU (β = 0.222 and p = 0.001), CUR (β = 0.135 and p = 0.001), JOY (β = 0.145 and p = 0.002), and ATT (β = 0.236 and p = 0.001) yielded positive and significant effects on IM. All HMSAM constructs were found significant compared to TPB constructs. This circumstance supported the substance of HMSAM's original framework. Particularly, PU, CUR, JOY, and IM constructs are part of the original HMSAM. However, other studies overlooked the connection between PU and IM [15,16]. In this study, NFT gamers put value in the play-to-earn model. Since it was deemed useful for the users, they found the games immersive. Additionally, NFT gamers were engaged in the NFT gaming world without minding the distractions as long as the games kindle their minds and give pleasure. The significant connections of CUR and JOY with IM also had similar results to the past studies [15,16]. Although only one TPB construct influenced IM, ATT had the highest direct coefficient among all constructs influencing IM. Surprisingly, NFT gamers' attitudes held great assistance to increase immersion in playing games. Gamers who were keen on playing the game were most likely to engage in NFT games as they feel absorbed by the game's purpose and interactive interface. In a similar study, gamers were immersed because of the game's contents and visions [46]. These features enhanced gamers' analysis skills.

Meanwhile, SN and PBC did not yield a significant influence on IM. Subjective norm involves obtaining information from other users and most NFT gamers did not feel too confident to trust in others' suggestions. Thereby, people surrounding the respondents lacked an effect on respondents' immersion in the game. Since engaging in NFT games needs cryptocurrency knowledge, users are expected to do their own research. NFT gamers were not fully absorbed by the game as they need to shell out finances. Contrary to the findings of Esteves et al. [47], influences from other gamers and online communities increased users' engagement and trust in online games. Result differences occurred because the current study assessed NFT games that would require users to buy game characters before they could play the game. Meanwhile, the past study assessed free-to-play online games. On the other hand, it was an unexpected result when PBC and IM failed to have a significant connection as opposed to the conclusion of Lowry et al. [16]. This current study argued that NFT gamers’ behavioral control depends on cryptocurrency values. An example is the sudden price decrease of SLP cryptocurrency in Axie Infinity. If cryptocurrency value decreases, NFT gamers would look for other games or forms of investment. Hence, most players were waiting for the SLP value increase. As it continuously increases, people get immersed in the game as it is expected to yield profit and capital return.

Between BI and IM, BI (0.553) obtained a greater R-squared value compared to IM (0.406). In addition, several constructs significantly influenced BI more than IM. This result implied that BI was best explained by HMSAM and TPB constructs. Users were more inclined to continue using NFT games for a long period. They also intend to maximize the NFT game's model as a source of income. Although they valued the financial aspect better, NFT gamers still found the games entertaining and immersive.

### 5.2 Theoretical contributions

Two theories (HMSAM and TPB) were combined to analyze the factors affecting NFT gamers' intended and immersive behaviors. Furthermore, the researchers supplemented the theory with a separate hedonic motivation construct that strengthened the influences on HMSAM and TPB original constructs. Additional connections were also merged into the model to expand the understanding of the proposed SEM framework. As of this writing, the integration of HMSAM and TPB in NFT gaming was overlooked by other researchers. The present study supported the significant influences of several constructs on each other. Overall, this study discussed the significance of PEOU, SN, HM, PU, CUR, JOY, ATT, PBC, BI, and IM in analyzing NFT gamers’ behaviors. Future researchers could adapt the modified SEM and further improve them as some hypotheses lacked significance. The proposed framework was not limited to NFT gaming, but could also be utilized in other concepts. Particularly, HMSAM and TPB could be adapted to any digital context. For instance, researchers could use the combined theories to explore digital transformation, improve the current digital systems, and understand the impact of digital applications on users or stakeholders.

### 5.3 Practical implications

NFT gamers valued perceived ease of use. This construct was indirectly influenced by hedonic motivation and directly influenced by subjective norm. Thus, the researchers encouraged NFT developers to create simplified instructions that could be followed by users easily. NFT developers should also update the games and perform system quality checks frequently to eliminate bugs. These bugs are system errors, which prevent users to play games seamlessly. Additionally, NFT stakeholders must put significance in the game branding to spread its influence on online communities. Since NFT gamers engage with other gamers through online platforms, NFT stakeholders should value digital marketing in streaming platforms (e.g., YouTube, Facebook, Discord, Twitch, and Steam).

Hedonic motivation, curiosity, and joy were taken into consideration by ensuring that the games offer excitement. NFT stakeholders should perform market research and establish game challenges based on trends and demographics. They must combine users’ personal interests with the current trend to make NFT game challenges immersive. This technique ensures that the games are up-to-date and innovative. Moreover, NFT gamers disliked extremely automated interfaces where they could no longer use their minds to think. NFT developers should steer away from using several automation features because the gamers wanted to feel challenged and see that their hard work is paying off.

Furthermore, perceived usefulness was a significant construct influencing NFT gamers' intention and immersive behavior. NFT games were deemed useful because of the play-to-earn model. Since gamers wanted to earn money and enjoy the game, their first goal was to gain a return on investment, next to gaining additional profit, followed by the pleasure of playing the game. NFT developers should monitor SLP values since gamers earn money through SLP increases. It should also be noted that quick tutorials must be given to new gamers since not everyone is aware of the relationships between NFT games and SLP cryptocurrency. NFT developers should also provide cryptocurrency news to tenured gamers since they tend to neglect current news. These approaches also addressed the user's attitude and perceived behavioral control, which in turn affected the user's intention and immersive behavior based on the corresponding significant hypotheses.

### 5.4 Limitations and recommendation

The study accomplished its objectives and discussed substantial findings, but the researchers acknowledged its limitations. First, four hypotheses were found insignificant. Also, twenty-one out of twenty-four hypotheses were found significant, which exposed that 87.5% of the hypotheses were supported by the modified framework. Second, NFT gamers were not segmented into the type of games they played. In this study, researchers only asked about the NFT games that the respondents played. However, the respondents were not assessed based on those games independently. Nonetheless, the study approached NFT gamers' behaviors holistically. Third, the study could be expanded by disseminating questionnaires onsite. This study utilized online forms due to the COVID-19 risks and restrictions. The online survey process enables the researcher to conduct the data collection much faster and with larger sample size, however it will improve the data collection's reliability if the researcher would have the ability to conduct collection face-to-face. Nevertheless, the current study employed reliability tests, which proved the data consistency.

Through the identified limitations of the study the researcher would recommend the following improvements that can be done on the study. First, the failed hypotheses could be developed by supplementing the corresponding constructs with new indicators from other references. Although they were deemed insignificant, the study discussed arguments supporting the hypotheses' insignificance. As for future researchers, it would be much better to improve the indicators of the four insignificant hypotheses. Second, future researchers could explore the different intentions and immersive behaviors among Axie Infinity, Mir4, and Pegaxy users. In this manner the researcher's novelty would allow them to assess the difference of behavior of users towards the different popular games present on the industry. Hence, creating more specific research. Lastly, disseminating questionnaires in a face-to-face arrangement, researchers could explore respondents that were outside their social circles. These limitations could be used by other researchers to improve the study further.

## 6 Conclusion

As the popularity of NFT games in society starts to increase, understanding the new concept of game-based earning must be studied. Thus, understanding the behavior of NFT gamers was conducted to determine the possible factors of engagement towards it. This study utilized SEM techniques to scrutinize all hypotheses surrounding HMSAM and TPB. A total of 1082 respondents were gathered. Their responses underwent data validity and model fit tests. The final SEM was modified after filtering indices that met the required cut-off.

Among the twenty-four (24) hypotheses, twenty-one (21) hypotheses were supported by the study. Hedonic motivation significantly affected all the HMSAM and TPB constructs. Meanwhile, a TPB construct (subjective norm) influenced perceived ease of use, which eventually influenced a few HMSAM constructs (perceived usefulness and joy) and all TPB constructs. These hypothesized relationships supported the integration of HMSAM and TPB. Moreover, perceived behavioral control had the highest effect on the behavioral intentions of users toward NFT games. This was followed by curiosity, attitude, joy, and perceived usefulness, arranged in the highest to lowest direct effect. On the other hand, attitude had the highest influence on immersive behavior felt by NFT gamers. Also, perceived usefulness, joy, and curiosity significantly affected immersion; these constructs were ranked from 2nd to 4th highest direct coefficient. The researchers concluded that varying factors affect NFT gamers’ intention in playing NFT games for a long period and immersive behavior when playing the game.

This research presented theoretical contributions and practical implications to contribute to the academe and gaming industry. The academe industry, including researchers, could maximize the modified SEM, findings, and limitations. This study was not limited to NFT gaming since the framework could be utilized in all digital contexts. Furthermore, NFT stakeholders, such as management, businessmen, investors, and developers, could adopt the presented managerial implications to keep NFT gamers engaged in playing NFT games. They could also modify the findings based on their target market.

## Author contribution statement

William Davin D. Perez: Conceived and designed the experiments; Performed the experiments; Analyzed and interpreted the data; Contributed reagents, materials, analysis tools or data; Wrote the paper. Maela Madel L. Cahigas; Michael Nayat Young; Reny Nadlifatin: Analyzed and interpreted the data; Wrote the paper. Yogi Tri Prasetyo: Conceived and designed the experiments; Performed the experiments; Analyzed and interpreted the data; Wrote the paper. Satria Fadil Persada: Conceived and designed the experiments; Analyzed and interpreted the data.

## Data availability statement

Data will be made available on request.

## Funding statement

This research was funded by Mapúa University Directed Research for Innovation and Value Enhancement (DRIVE).

## Declaration of competing interest

The authors declare that they have no known competing financial interests or personal relationships that could have appeared to influence the work reported in this paper.
